# The profile of chiropractors managing patients with low back-related leg pain: analyses of 1907 chiropractors from the ACORN practice-based research network

**DOI:** 10.1186/s12998-019-0239-x

**Published:** 2019-04-17

**Authors:** Matthew Fernandez, Craig Moore, Wenbo Peng, Katie de Luca, Katherine A. Pohlman, Michael Swain, Jon Adams

**Affiliations:** 1 Department of Chiropractic, Faculty of Science and Engineering, Macquarie University, Level 3, Room 369, 17 Wally’s Walk, Sydney, NSW Australia; 2 Chiropractic Academy for Research Leadership (CARL), Sydney, Australia; 3 Faculty of Health, University of Technology Sydney, Sydney, NSW Australia; 4 Research Institute, Parker University, Dallas, Texas USA

**Keywords:** Low back pain, Leg pain, Referred, Radicular, Chiropractic, Chiropractor, Practice-based research network

## Abstract

**Background:**

Approximately 60% of people with low back pain also have associated leg pain symptoms. Guidelines for low back pain recommend non-pharmacological approaches, including spinal manipulation - a therapy provided by chiropractors. However, limited empirical data has examined the characteristics of chiropractors managing patients with low back-related leg pain (LBRLP). Our objective is to describe the prevalence, profile and practice characteristics of Australian chiropractors who often treat LBRLP, compared to those who do not often treat LBRLP.

**Methods:**

This is a cross-sectional analysis of a nationally representative sample from the Australian Chiropractic Research Network (ACORN). This study investigated the demographic and practice characteristics as well as clinical management of chiropractors who ‘often’ treated patients with LBRLP compared to those who treated LBRLP ‘never/rarely/sometimes’. Multiple logistic regression models identified independent factors associated with chiropractors who ‘often’ treated patients with LBRLP.

**Results:**

A total of 1907 chiropractors reported treating patients experiencing LBRLP, with 80.9% of them ‘often’ treating LBRLP. Chiropractors who ‘often’ treated LBRLP were more likely to manage patients with multi-site pain including axial low back pain (OR = 21.1), referred/radicular neck pain (OR = 10.8) and referred/radicular thoracic pain (OR = 3.1). While no specific management strategies were identified, chiropractors who ‘often’ treated LBRLP were more likely to discuss medication (OR = 1.8), manage migraine (OR = 1.7) and degenerative spine conditions (OR = 1.5), and treat women during pregnancy (OR = 1.6) and people with work-related injuries (OR = 1.5), compared to those not treating LBRLP frequently.

**Conclusions:**

Australian chiropractors frequently manage LBRLP, although the nature of specific management approaches for this condition remains unclear. Further research on the management of LBRLP can better inform policy makers and educators interested in upskilling chiropractors to deliver safe and effective treatment of LBRLP.

## Background

Approximately 60% of people who present to primary care with low back pain, also report leg pain due to dysfunctional musculoskeletal or neural structures [1]. However, distinguishing between referred and radicular pain is a clinical challenge [2]. Leg pain caused by ligament, joint or disc structures of the low back is generally classified as referred pain while radicular pain is associated with nerve root compression, largely caused by a herniated intervertebral lumbar disc [3]. There is substantial variation in the prevalence of radicular low back pain, with estimates ranging from 1.6 to 43% [4]. These variations likely reflect the numerous criteria, definition and assessment methods relating to referred and radicular symptoms in the peer-reviewed literature [5]. For consistency we here employ the terms “referred” and “radicular” to signify low back-related leg pain (LBRLP).

There is a lack of long-term positive effects for the management of LBRLP with respect to prescription medication [6, 7], epidural corticosteroid injections [8] and surgery [9]. While non-pharmacological treatments are preferred, high-quality evidence for the effectiveness of these therapies is scarce. For instance, traction as a single treatment or in combination with physiotherapy, has little to no effect on LBRLP [10]. Exercise is an effective modality compared to usual care [11], and evidence generally supports spinal manipulation compared to sham, no treatment, passive modalities, education and exercise in the management of LBRLP [12]. A more recent high-quality, multimodal randomized controlled trial, which included spinal manipulation together with home exercise and advice as the experimental group, was superior for LBRLP in the short-term, compared to exercise and advice alone in the control group [13].

In Australia chiropractors are a common health provider for those with low back pain [14, 15]. In addition to spinal manipulation [16, 17], chiropractors adopt a multimodal approach, incorporating a number of conservative therapies like soft tissue treatments and exercise prescription [18, 19], when managing low back pain [20]. Despite these treatment approaches, there is at present, limited information on the clinical characteristics of chiropractors that frequently manage LBRLP. In response, this study aimed to investigate: 1) the proportion of Australian chiropractors who regularly treat patients who present with lumbar pain and associated referred or radicular symptoms; and 2) the practitioner, clinical practice and clinical management characteristics factors associated with those chiropractors who frequently manage patients who present with lumbar pain and associated referred or radicular symptoms.

## Methods

### Study design and setting

Details of the Australian Chiropractic Research Network (ACORN) project recruitment and participating sample have been outlined elsewhere [21, 22]. Briefly, ACORN - a national practice-based research network (PBRN), recruited registered Australian chiropractors for a national-wide cross-sectional survey, between March and July 2015. All 4684 chiropractors registered in Australia at the time of recruitment were invited to participate and received an invitation pack (information sheet, consent form and a 21-item practitioner questionnaire) by post or email. Chiropractors were invited to complete the practitioner questionnaire in either hard copy or online (SurveyGizmo™). The ACORN survey attracted a completion (response) rate of 43% (*n* = 2005). The ACORN participants constitute a representative sample of the wider Australian chiropractic population in terms of age, gender, and practice location when compared to national registration records at the time of recruitment [23]. Ethics approval of the ACORN project was obtained from the Human Research Ethics Committee of the University of Technology Sydney (#2014000027).

### Variables and measurements

The practitioner survey instrument collected information in an attempt to describe chiropractic practice in three key areas. The first was Australian practitioner characteristics comprising of: age, gender, professional qualifications and years in private practice. The second area was clinical practice characteristics, including: average patient care hours, average number of weekly patient visits, practice location, types of health professionals working in the chiropractor’s practice location, professional referral relationships and use of diagnostic imaging. The third area was clinical management, divided into four sub-sections and based on a four-point frequency scale (‘never’, ‘rarely’, ‘sometimes’ and ‘often’) and included: frequency with which the chiropractors discuss listed aspects of health promotion in their care plans; frequency with which the chiropractors treat patients presenting with a range of conditions; frequency with which chiropractors treat certain patient subgroups as listed; and the frequency with which the chiropractors utilise certain treatment techniques/musculoskeletal interventions.

Only participants who completed the clinical management question with regards to the *frequency* of treating referred/radicular low back pain within the ACORN practitioner questionnaire were included in this study. The ACORN study therefore represents general chiropractic practice (and not behaviours specific to the management of LBRLP). The response options to the people with referred/radicular low back pain (i.e., LBRLP) include ‘never’, ‘rarely’, ‘sometimes’ and ‘often’, which were merged into two categories ‘never/rarely/sometimes’ and ‘often’ for analyses and interpretation.

### Statistical methods

Practitioner characteristics, clinical practice characteristics and clinical management were compared between chiropractors ‘never/rarely/sometimes’ treating LBRLP and those ‘often’ treating LBRLP. Statistical analyses were conducted by the software Stata 13.1. Bivariate analyses were performed via parametric tests and non-parametric tests according to the normality of each variable. A backward stepwise logistic regression was used to identify the most important predictors of chiropractors who are ‘often’ treating LBRLP compared to those not ‘often’ treating LBRLP. All variables associated with the LBRLP treatment through bivariate analyses at a *p*-value of ≤0.25 were entered into the regression model [24] and variables were removed from the model if their corresponding coefficient had a p-value> 0.05. Odds ratios (OR) of the identified predictors were reported with 95% confidence intervals (95% CI), based on robust standard error estimators.

## Results

A total of 1907 (95.1%) ACORN participants included in this present study indicated that they treated patients presenting with LBRLP, and 80.9% (*n* = 1543) of these participants reported ‘often’ treating LBRLP. Table 1 shows the comparisons regarding chiropractic practitioner characteristics between those who ‘often’ treat LBRLP and those who did not often treat LBRLP. There was no statistically significant difference between those ‘often’ and not often treating LBRLP regarding age, gender, qualifications and years in practice. The average age of chiropractors who often treated LBRLP was 42.2 (SD: 12.1) with 62.9% being male and an average of 15.9 (SD: 11.3) years in private chiropractic practice. The highest level of chiropractic professional qualification for the overall ACORN study sample was most often Bachelor degree (34.6%) and Master’s degree (32.7%) while only 0.9% of the participated chiropractors have obtained PhD. Similarly, Bachelor degree (35.4%) and Master’s degree (31.4%) were also the most common highest qualifications for chiropractors ‘often’ treating LBRLP, followed by a Doctor of Chiropractic (29.8%), Diploma (2.7%), and PhD (0.7%). Chiropractors who ‘often’ treated LBRLP reported significantly more patient visits per week on average (mean ± SD: 90.8 ± 57.7) and significantly more patient care hours per week on average (mean ± SD: 28.2 ± 16.6), compared to those not often treating LBRLP.

**Table 1:** Chiropractors’ characteristics regarding the frequency of treating patients with low back-related leg pain

Characteristics	Never/rarely/ sometimes (*n* = 364)	Often (n = 1543)	
			p
Gender *n (%)*			0.549
Male	221 (61.2%)	967 (62.9%)	
Female	140 (38.8%)	570 (37.1%)	
Qualification *n (%)*			0.691
Diploma	14 (3.9%)	42 (2.7%)	
Bachelor	122 (33.8%)	542 (35.4%)	
Doctor of Chiropractic	104 (28.8%)	456 (29.8%)	
Masters	117 (32.4%)	480 (31.4%)	
PhD	4 (1.1%)	11 (0.7%)	
	Mean (SD)	Mean (SD)	
Age (in years)	42.4 (12.1)	42.2 (12.1)	0.707
Years in private chiropractic practice	15.9 (11.5)	15.9 (11.3)	0.946
Patient care hours/week	24.6 (10.9)	28.2 (16.6)	< 0.001
Patient visits/week	73.4 (54.1)	90.8 (57.7)	< 0.001

*SD* standard deviation, *P* p-value

The chiropractors’ practice characteristics regarding the frequency of treating LBRLP are presented in Table 2. Of those chiropractors who ‘often’ treated LBRLP, 1143 (74.2%) practiced in only one location and 1093 (75.3%) practiced in urban areas exclusively, which were not statistically significantly different than those chiropractors not often treating LBRLP. Chiropractors who ‘often’ treated LBRLP were more likely to work with another chiropractor in their practice location, compared to those who not often treated this condition (*p* = 0.004). In addition, chiropractors who ‘often’ treated LBRLP were more likely to hold referral relationships with podiatrists (*p* < 0.001), physiotherapists (*p* = 0.011), and/or exercise physiologist (*p* = 0.039) than chiropractors who not often treated LBRLP. Chiropractors ‘often’ treating LBRLP were significantly more likely to use diagnostic imaging as part of their practice, compared to those who not often treating this condition (*p* < 0.001).

**Table 2:** Chiropractors’ practice characteristics regarding the frequency of treating patients with low back-related leg pain

Characteristic	Never/rarely/ sometimes (n = 364)	Often (n = 1543)	
			p
Practice location *n (%)*			
Urban	271 (79.5%)	1093 (75.3%)	0.102
One location only	280 (77.6%)	1143 (74.2%)	0.188
Other practitioners in the same practice location *n (%)*			
General practitioner	25 (6.9%)	98 (6.4%)	0.718
Podiatrist	35 (9.6%)	146 (9.5%)	0.928
Medical specialist	10 (2.8%)	42 (2.7%)	0.979
Physiotherapist	30 (8.2%)	148 (9.6%)	0.426
Another chiropractor	189 (51.9%)	929 (60.2%)	0.004
Exercise physiologist	17 (4.7%)	108 (7.0%)	0.106
Psychologist/Counsellor	51 (14.0%)	189 (12.3%)	0.362
Occupational therapist	7 (1.9%)	41 (2.7%)	0.421
Referral relationships with other practitioners *n (%)*			
General practitioner	194 (53.3%)	890 (57.7%)	0.129
Podiatrist	115 (31.6%)	644 (41.7%)	< 0.001
Medical specialist	62 (17.0%)	244 (15.8%)	0.568
Physiotherapist	95 (26.1%)	509 (33.0%)	0.011
Exercise physiologist	44 (12.1%)	254 (16.5%)	0.039
Psychologist/Counsellor	55 (15.1%)	217 (14.1%)	0.608
Occupational therapist	33 (9.1%)	125 (8.1%)	0.548
Using diagnostic imaging (used often) *n (%)*	112 (31.0%)	787 (51.3%)	< 0.001

*P* p-value

Table 3 reports the clinical management of chiropractors who treated LBRLP. Several statistically significant differences were found between those chiropractors who ‘often’ treated LBRLP and those who did not often treat LBRLP for several components related to their care/management plans. For instance, chiropractors who ‘often’ treated LBRLP were more likely to discuss diet and nutrition (*p* < 0.001), cigarette smoking, drugs and alcohol (*p* = 0.011), physical activity and fitness (p < 0.001), occupational health and safety (p < 0.001), pain counselling (p < 0.001), nutritional supplements (*p* = 0.004), and/or medications (p < 0.001) with their patients, compared to those who did not often treat LBRLP. Moreover, chiropractors ‘often’ treating LBRLP were more likely to treat patients with neck pain (axial and referred/radicular), thoracic pain (axial and referred/radicular), low back pain (axial), lower limb musculoskeletal disorders, upper limb musculoskeletal disorders, postural disorders, degenerative spine conditions, headache disorders, migraine disorders, spine health maintenance/prevention, and/or non-musculoskeletal disorders, compared to those not often treating LBRLP (all *p* ≤ 0.005).

**Table 3:** Chiropractors’ clinical management characteristics regarding the frequency of treating patients with low back-related leg pain

Characteristic	Never/rarely/ sometimes (n = 364)	Often (n = 1543)	
			p
Management plan (discussed often) *n (%)*			
Diet/Nutrition	152 (42.2%)	810 (52.6%)	< 0.001
Smoking/Drugs/Alcohol	71 (19.7%)	401 (26.2%)	0.011
Physical activity/Fitness	282 (77.9%)	1335 (87.0%)	< 0.001
Occupational health and safety	116 (32.1%)	659 (43.2%)	< 0.001
Pain counselling	63 (17.6%)	403 (26.6%)	< 0.001
Nutritional supplements (including vitamins, minerals, herbs)	110 (30.6%)	597 (38.8%)	0.004
Medications (including for pain/inflammation)	44 (12.2%)	392 (25.8%)	< 0.001
Conditions (treated often) *n (%)*			
Neck pain: Axial	275 (75.8%)	1509 (97.9%)	< 0.001
Neck pain: Referred/Radicular	35 (9.6%)	1162 (75.3%)	< 0.001
Thoracic pain: Axial	226 (62.4%)	1382 (90.0%)	< 0.001
Thoracic pain: Referred/Radicular	20 (5.6%)	857 (56.1%)	< 0.001
Low back pain: Axial	273 (75.2%)	1525 (99.2%)	< 0.001
Lower limb musculoskeletal disorders	135 (37.3%)	1013 (65.7%)	< 0.001
Upper limb musculoskeletal disorders	137 (37.7%)	1051 (68.5%)	< 0.001
Postural disorders (including lordosis, thoracic kyphosis, scoliosis)	147 (41.6%)	992 (65.9%)	< 0.001
Degenerative spine conditions (including spondylolisthesis)	134 (38.1%)	1070 (71.1%)	< 0.001
Headache disorders	229 (64.9%)	1396 (92.5%)	< 0.001
Migraine disorders	77 (21.8%)	910 (60.3%)	< 0.001
Spine health maintenance/prevention	200 (56.7%)	1158 (76.9%)	< 0.001
Non-musculoskeletal disorders	57 (22.7%)	354 (31.6%)	0.005
Patient groups (treated often) *n (%)*			
Older people (65 years or over)	196 (54.4%)	1197 (78.0%)	< 0.001
Aboriginal and Torres Strait Islander people	4 (1.1%)	29 (1.9%)	0.303
Pregnant women	81 (22.5%)	613 (40.1%)	< 0.001
Athletes or sports people	115 (32.1%)	819 (53.7%)	< 0.001
People with work-related injuries	74 (21.3%)	594 (39.5%)	< 0.001
People with traffic-related injuries	18 (5.1%)	235 (15.7%)	< 0.001
People receiving post-surgical rehabilitation	11 (3.2%)	109 (7.3%)	0.005
Non-English speaking ethnic group(s)	9 (2.7%)	108 (7.4%)	0.002
Techniques/methods (used often) *n (%)*			
Drop-piece techniques/Thompson or similar	167 (46.7%)	843 (55.6%)	0.002
Biomechanical pelvic blocking/Sacro-Occipital technique	138 (38.3%)	684 (45.3%)	0.017
Instrument adjusting	170 (47.4%)	810 (53.3%)	0.043
Chiropractic BioPhysics	14 (4.1%)	64 (4.4%)	0.766
High velocity, low amplitude adjustment/manipulation/mobilisation	265 (73.8%)	1283 (84.4%)	< 0.001
Applied kinesiology	51 (14.4%)	246 (16.5%)	0.343
Flexion-distraction	15 (4.2%)	132 (8.9%)	0.004
Functional neurology	35 (10.0%)	209 (14.2%)	0.040
Extremity manipulation	169 (47.1%)	939 (61.8%)	< 0.001
Musculoskeletal interventions (used often) *n (%)*			
Dry needling or acupuncture	40 (11.2%)	219 (14.4%)	0.110
Soft tissue therapy, trigger point therapy, massage therapy, stretching	218 (60.4%)	1036 (67.7%)	0.008
Electro-modalities (TENS, laser, interferential/ultrasound therapy)	25 (6.9%)	158 (10.4%)	0.047
Heat/Cryotherapy	40 (11.1%)	273 (18.0%)	0.002
Orthotics	22 (6.1%)	169 (11.2%)	0.005
Specific exercise therapy/rehabilitation/injury taping	155 (43.2%)	770 (50.8%)	0.009

*P* p-value

As shown in Table 3, chiropractors who ‘often’ treated LBRLP were more likely to treat people aged over 65 years, pregnant women, athletes or sports people, people with work-related injuries, people with traffic-related injuries, people receiving post-surgical rehabilitation, and/or people from non-English speaking ethnic group(s) (all p ≤ 0.005). Regarding management techniques/methods utilised by chiropractors, those who ‘often’ treated LBRLP more often used drop-piece techniques/Thompson or similar (*p* = 0.002), biomechanical pelvic blocking/Sacro-Occipital technique (*p* = 0.017), instrument adjusting (*p* = 0.043), high-velocity, low-amplitude adjustment/manipulation/mobilisation (*p* < 0.001), flexion distraction (*p* = 0.004), functional neurology (*p* = 0.040), and/or extremity manipulation (p < 0.001). Also, compared to chiropractors who did not often treat LBRLP, those who ‘often’ treated LBRLP were more likely to employ musculoskeletal interventions including soft tissue therapy, trigger point therapy, massage therapy, stretching (*p* = 0.008), electro-modalities (*p* = 0.047), heat/cryotherapy (*p* = 0.002), orthotics (*p* = 0.005), and/or specific exercise therapy/rehabilitation/injury taping (*p* = 0.009).

The results of the logistic regression analysis is demonstrated in Table 4. Factors independently associated with chiropractors who more ‘often’ treat patients with LRLBP include: frequently discuss medications as part of the care/management plan (OR: 1.77; 95% CI: 1.14–2.73), frequently treat referred/radicular neck pain (OR: 10.81; 95% CI: 6.63–17.63), frequently treat referred/radicular thoracic pain (OR: 3.14; 95% CI: 1.72–5.71), frequently treat axial low back pain (OR: 21.08; 95% CI: 10.10–44.00), frequently treat degenerative spine conditions (OR: 1.53; 95% CI: 1.11–2.11), and/or frequently treat migraine disorders (OR: 1.70; 95% CI: 1.18–2.43). Additionally, chiropractors who ‘often’ treated LBRLP were more likely to treat pregnant women (OR: 1.58; 95% CI: 1.08–2.30) and people with work-related injuries (OR: 1.49; 95% CI: 1.04–2.14).

**Table 4:** Logistic regression analysis identifying predictors for chiropractors who often treat patients with low back-related leg pain as compared to those who do not

Factors	Odds Ratio	95% CI	P
Axial low back pain			< 0.001
Never/rarely/sometimes	Reference	1.00	
Often	21.08	10.10, 44.00	
Referred/radicular neck pain			< 0.001
Never/rarely/sometimes	Reference	1.00	
Often	10.81	6.63, 17.63	
Referred/radicular thoracic pain			< 0.001
Never/rarely/sometimes	Reference	1.00	
Often	3.14	1.72, 5.71	
Discussing medications			0.010
Never/rarely/sometimes	Reference	1.00	
Often	1.77	1.14, 2.73	
Migraine disorders			0.004
Never/rarely/sometimes	Reference	1.00	
Often	1.70	1.18, 2.43	
Pregnant women			0.017
Never/rarely/sometimes	Reference	1.00	
Often	1.58	1.08, 2.30	
Degenerative spine conditions			0.010
Never/rarely/sometimes	Reference	1.00	
Often	1.53	1.11, 2.11	
People with work-related injuries			0.030
Never/rarely/sometimes	Reference	1.00	
Often	1.49	1.04, 2.14	

## Discussion

Our study identifies several clinical management differences among chiropractors who ‘often’ treat LBRLP compared to those who do not often treat this condition. One finding is that those chiropractors who ‘often’ treat LBRLP, are more likely to discuss medication for pain and inflammation with patients. Given the more intense pain and disability associated with LBRLP, compared to non-specific low back pain [25], this finding is not unexpected. Pain medication discussions may also be underpinned by the considerable side effects associated with numerous pharmacological interventions for LBRLP [7], and with uncertainty regarding medication efficacy and tolerability [6], major international clinical guidelines for non-specific low back and leg pain now discourage the use of analgesic medication for this condition [11, 26, 27]. Chiropractors presently do not have medication prescription rights in Australia [28], yet a baseline level of pharmacological understanding, such as medication usage and harmful interactions between different medications or adverse drug reactions [29] is essential knowledge for chiropractors [30], particularly in older people with LBRLP [31]. Importantly, many other factors may influence a chiropractor’s decision to discuss medication with patients that are not solely due to LBRLP. For instance, medications such as hypertensive drugs may influence chiropractic care [32], while others such as blood thinners, may pose a risk, for example an increased risk of bleeding and constitute important clinical information for chiropractors to ensure quality and safe patient care [33]. The specific nature of medication discussions of Australian chiropractors regarding LBRLP in this study were not examined and requires further examination in future research.

Our study also found that chiropractors who ‘often’ treat LBRLP are more likely to provide treatment to other spinal pain regions, including referred/radicular neck pain and referred/radicular thoracic pain (in addition to axial low back pain) than those chiropractors who do not often treat LBRLP. This finding may possibly be explained by research indicating that lumbar, thoracic and cervical spine pain are likely part of a general musculoskeletal pain syndrome rather than a distinct, site-specific complaint [34]. LBRLP is reportedly more prevalent and frequent in those who report pain at multiple musculoskeletal sites [35, 36]. Although the mechanism behind multiple co-occurring pain regions is unknown, low back and neck pain for instance, share many commonalities including similar one year prevalence rates [37, 38], poorer health [35] and increased comorbidity [39]. Chiropractors integrate several manual-based techniques in clinical practice [22], and our analyses suggests multi-region manual therapy may already be carried out by chiropractors managing LBRLP. Nevertheless, musculoskeletal guidelines generally focus on single site-specific complaints, hence a greater understanding of the management of multi-region spinal and coexisting musculoskeletal pain problems is required [40].

Chiropractors who ‘often’ treat LBRLP as shown in our study, will also often treat people with degenerative spine conditions. This finding was not unexpected, with degenerative spine conditions such as lumbar spinal stenosis (i.e., age-related central and/or lateral lumbar spinal canal narrowing) identified as a common cause of LBRLP and disability in older people [41].

There is a marked reduction in physical and mental quality of life, and a subsequent impact in older adults functional capacity, i.e., walking, due to degenerative LBRLP [42]. As a result, many older people utilize chiropractic services due to degenerative lumbar spinal complaints [43]. For instance, an Australian study found 13% of patient encounters were those aged 65 years and older [15], while an American study showed approximately 14% of chiropractic patients are over the age of 65 years, with low back pain the most common musculoskeletal complaint [43]. Despite this utilisation, there is a lack of high-quality evidence for the conservative management of lumbar spinal stenosis [41, 44]. Although a recent clinical trial showed good, short-term pain and functional outcomes [45], there is a greater need for more chiropractic research regarding the long-term effectiveness of optimal LBRLP treatment in people with degenerative spine conditions [20, 46, 47].

Chiropractors who ‘often’ treat LBRLP in this present study are more likely to provide treatment to pregnant women when compared to chiropractors not often treating LBRLP. Given the changes in centre of gravity, weight gain and ligamentous laxity throughout the pregnancy, labour, and post-partum periods [48], this finding is plausible. Women frequently seek chiropractic care for pregnancy, with 80% reporting low back pain or pelvic pain [49] or a combination of both [50]. Further, recent data has identified prevalence rates as high as 22% in pregnant women who experience LBRLP and seek chiropractic care [51]. Previous systematic reviews also report favourable effects regarding the efficacy of chiropractic treatment for pregnancy-related low back pain [49, 52, 53]. As emerging research indicates that a considerable proportion of pregnant women with LBRLP seek treatment [54], chiropractic appears to represent a substantial care option for this population group.

In this study, the treatment of people with work-related injuries are another factor independently associated with chiropractors ‘often’ treating LBRLP. This relationship may relate to the increased loading of lumbar spine structures [55], during physically strenuous or demanding workloads (i.e., whole body vibration, frequent bending, twisting and lifting), are known risk factors for both non-specific low back and LBRLP [56–60]. As such, work-related LBRLP complaints are common, placing a substantial economic burden on health-care services [61]. Hence, it is reasonable to expect workplace injury prevention-like strategies taking place within the chiropractic setting, by not only treating injured workers but discussing the modification of physical and psychosocial factors (i.e., patient fear and sense of vulnerability related to injury) to reduce injury risk and cost [62]. Structured exercise is one intervention shown to be protective in managing LBRLP in the short-term [63], while physical activity, i.e., walking and cycling [64], is also protective against work-related LBRLP [65]. While a national-based study found that 85% of Australian chiropractors report discussing physical activity and/or exercise interventions with patients [22], whether these discussions specifically target work-related injuries and LBRLP remains unclear.

## Limitations

Our study explores personal, practice and clinical characteristics of chiropractors who frequently treat LBRLP, identifying valuable questions for future investigation regarding the chiropractic management of this condition. However, there are limitations to our research that need to be considered. The ACORN practitioner questionnaire was designed to provide high-quality baseline data regarding the chiropractic workforce and not the management of specific clinical chiropractic conditions. Despite the ACORN survey attracting a response rate of 43%, it is one of the largest voluntary workforce samples in both chiropractic and allied health care to date [21]. The ACORN survey combined the terms “referred” and “radicular” low back pain to indicate LBRLP, yet most published guidelines on low back pain support the identification of patients with leg pain due to nerve root involvement [66]. There may also be the potential for recall bias, given the self-reported nature of our study.

## Conclusion

Nearly three-quarters of Australian chiropractors reported frequently managing LBRLP, yet the specific nature of both their analyses and management approaches related to LBRLP remains unclear. Further, in-depth research is required with respect to these topics to better inform policy-makers, practitioners, educators and patients regarding the safe and effective treatment of LBRLP by chiropractors amongst the range of health providers available for this condition.

## Abbreviations

ACORN: Australian Chiropractic Research Network
LBRLP: Low back-related leg pain
PBRN: practice-based research network
